# Rapid Discovery of Illuminating Peptides for Instant Detection of Opioids in Blood and Body Fluids

**DOI:** 10.3390/molecules24091813

**Published:** 2019-05-10

**Authors:** Shabnam Jafari, Yann Thillier, Yousif H. Ajena, Diedra Shorty, Jiannan Li, Jonathan S. Huynh, Bethany Ming-Choi Pan, Tingrui Pan, Kit S. Lam, Ruiwu Liu

**Affiliations:** 1 Department of Biochemistry & Molecular Medicine, University of California Davis, Sacramento, CA 95817, USA; shjafari@ucdavis.edu (S.J.); Yann.Thillier@umassmed.edu (Y.T.); yousifajena5620@gmail.com (Y.H.A.); dshorty@ucdavis.edu (D.S.); jshuynh@ucdavis.edu (J.S.H.); bmpan@ucdavis.edu (B.M.-C.P.); 2 Department of Biomedical Engineering, University of California Davis, Davis, CA 95616, USA; jnli@stanford.edu (J.L.); trpan@ucdavis.edu (T.P.)

**Keywords:** OBOC, combinatorial chemistry, opioid, drug screen, molecular rotor dye, high throughput screening, sensor chip

## Abstract

The United States is currently experiencing an opioid crisis, with more than 47,000 deaths in 2017 due to opioid overdoses. Current approaches for opioid identification and quantification in body fluids include immunoassays and chromatographic methods (e.g., LC-MS, GC-MS), which require expensive instrumentation and extensive sample preparation. Our aim was to develop a portable point-of-care device that can be used for the instant detection of opioids in body fluids. Here, we reported the development of a morphine-sensitive fluorescence-based sensor chip to sensitively detect morphine in the blood using a homogeneous immunoassay without any washing steps. Morphine-sensitive illuminating peptides were identified using a high throughput one-bead one-compound (OBOC) combinatorial peptide library approach. The OBOC libraries contain a large number of random peptides with a molecular rotor dye, malachite green (MG), that are coupled to the amino group on the side chain of lysine at different positions of the peptides. The OBOC libraries were then screened for fluorescent activation under a confocal microscope, using an anti-morphine monoclonal antibody as the screening probe, in the presence and absence of free morphine. Using this novel three-step fluorescent screening assay, we were able to identify the peptide-beads that fluoresce in the presence of an anti-morphine antibody, but lost fluorescence when the free morphine was present. After the positive beads were decoded using automatic Edman microsequencing, the morphine-sensitive illuminating peptides were then synthesized in soluble form, functionalized with an azido group, and immobilized onto microfabricated PEG-array spots on a glass slide. The sensor chip was then evaluated for the detection of morphine in plasma. We demonstrated that this proof-of-concept platform can be used to develop fluorescence-based sensors against morphine. More importantly, this technology can also be applied to the discovery of other novel illuminating peptidic sensors for the detection of illicit drugs and cancer biomarkers in body fluids.

## 1. Introduction

Opioid abuse is a rapidly growing epidemic, and according to data from the US Department of Health and Human Services, it caused more than 130 deaths per day in the US alone in 2017. Since the year 2000, the rate of deaths from drug overdoses has increased by 137%, including a 200% increase in the rate of overdose deaths involving opioids (specifically opioid pain relievers and heroin) [1,2,3]. There is a medical need to develop a portable point-of-care diagnostic device that allows paramedics to rapidly identify the opioid drug(s) taken by unconscious patients, thereby allowing treatment to begin in the field, and effectively saving more lives. Current approaches for opioid identification and quantification in body fluids include immunoassay and chromatographic methods (e.g., LC-MS, GC-MS), which require expensive instrumentation and extensive sample preparations [4,5,6,7,8,9,10]. The current homogeneous enzymatic immunoassays do not identify the opioid and often give false positive results if the patients are taking standard therapeutic doses of some antibiotics [11,12]. We proposed developing a straightforward and innovative fluorescence-based sensor chip to sensitively detect opioid drugs in the blood, using a homogeneous immunoassay without any washing steps. Illuminating peptides that specifically interact with a selected monoclonal antibody (mAb) can be discovered via high throughput screening of the one-bead one-compound (OBOC) combinatorial peptide library [13], that is covalently linked to a molecular rotor dye (MRD). Once identified and immobilized as peptide microarrays on a chip, the generated illuminating peptide spots function as activatable fluorescent probes to monitor the physical interactions with its target protein, which in this study was the anti-morphine mAb.

The term, molecular rotor, refers to a class of twisted intramolecular charge transfer complexes (TICT), with photophysical characteristics that depend on their environment. This class of fluorophores is characterized by their formation of twisted states through the rotation of various segments of the structure with respect to the rest of the molecule. The aforementioned intramolecular rotation changes the ground state and the excited-state energies, whilst the molecular rotors de-excited from the twisted state either through emission of a red-shifted emission band or by non-radiative relaxation. Since this rotation and thus the twisted state formation rate are strongly dependent on the solvent, parameters such as solvent polarity, hydrogen bond formation, isomerization, and steric hindrance, which are the predominant forms of the solvent–fluorophore interactions, can all affect the emission properties. Of these, steric hindrance is of high importance because it links the solvents micro-viscosity to the formation rate of the twisted intramolecular charge transfer state, which in turn determines the spectral emission [14,15,16]. Molecular rotor dyes (MRDs) have become increasingly popular as molecular imaging probes, since they exhibit very unique properties and they offer the possibility of imaging target biomolecules with minimal background [17,18]. MRDs have already been used as probes for ligand-receptor binding, guest fluorophores that turn on fluorescence with a target RNA aptamer or labels of biomolecules [19,20,21]. 

**Figure 1:**
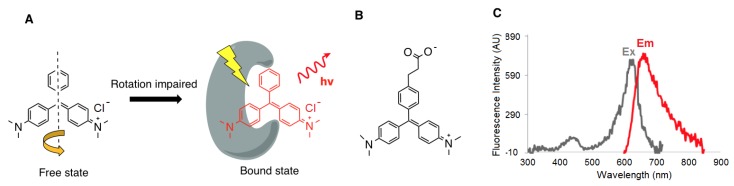
(**A**) Molecular rotor dye (MRD) malachite green (MG) in free and bound states. (**B**) Structure of the carboxyethyl malachite green (CEMG). (**C**) CEMG excitation, emission spectra were obtained in glycerol:PBS (80:20, *v*/*v*) at pH 7.4.

Malachite green (MG) is a triarylmethane MRD that has been extensively used in live cell imaging, as it exhibits low fluorescence in its unbound state, but has very high fluorescence (>2000-fold) when bound specifically to macromolecules (Figure 1A) [22,23]. The fluorescent-activation property of MG makes it an ideal candidate for the development of illuminating peptide-dye conjugates, which enables the dynamic detection of target–ligand interactions in the near infra-red range, whilst minimizing the background autofluorescence. Covalently attached molecular rotors are a widely applicable tool to study supramolecular protein assembly, and they can reveal the microrheological features of aggregating protein systems both in vitro and in cellulo, which are not otherwise observable through classical fluorescent probes operating in the light switch mode [24]. However, it is important to emphasize that the direct conjugation of MRDs to known protein ligands has not been well explored. To date, there have been few reports in the literature on using MRD-conjugated ligands to probe living cells. An example is using covalently attached molecular rotors as fluorogenic probes for monitoring peptide binding to class II MHC proteins in living cells [25]. A series of novel fluorogenic probes incorporating 4-*N*,*N*-dimethylaminophthalimidoalanine and 6-*N*,*N*-dimethylamino-2-3-naphthalimidoalanine have been developed to visualize these processes. These fluorophores show large changes in the emission spectra upon binding to class II MHC proteins. Peptides incorporating these fluorophores bind specifically to the class II MHC proteins on antigen-presenting cells and can be used to follow peptide binding in vivo. These probes have been used to track a developmentally regulated cell-surface peptide-binding activity in primary human monocyte-derived dendritic cells [25]. Another example is the successful imaging of the oxytocin G receptor using carbetocin peptide linked to an MRD [19]. MRDs have also been used as an exogenous probe to detect a single chain antibody against MG, that has been genetically grafted to the host protein for protein tracking in living cells [26]. 

**Figure 2:**
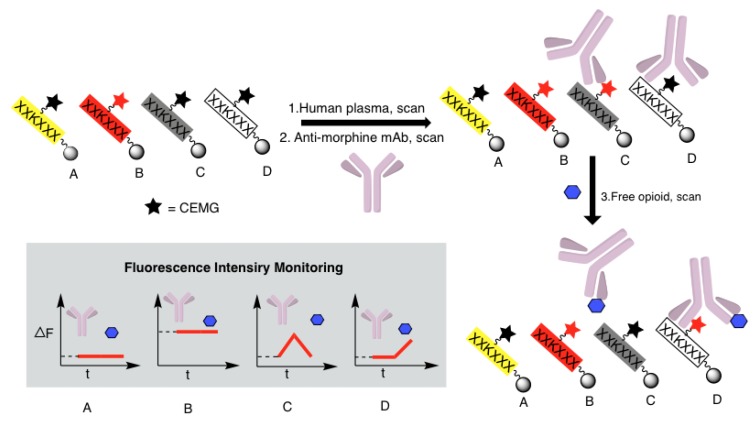
Three-step fluorescent screening assay of the illuminating peptide libraries.

The OBOC combinatorial library method has proven to be a powerful tool for identifying bioactive molecules, including peptides, peptidomimetics, and small molecules [13,27]. Using a “split-and-pool” solid-phase synthesis strategy [13,28], the OBOC combinatorial libraries can be rapidly prepared such that each bead displays 10^13^ copies of a single chemical entity [13]. This approach allows millions of compounds to be synthesized and screened simultaneously within a few weeks. Solid-phase peptide synthesis (SPPS), which allows for a rapid peptide synthesis on solid support with high efficiency and reduced side reactions [13,28,29], was employed in the synthesis of OBOC illuminating peptide libraries. These OBOC combinatorial libraries contain a large number of random peptides while incorporating a MRD, carboxyethyl malachite green (CEMG, Figure 1B), to the lysine side chain amino group at different positions of the peptide sequences. A subsequent three-step fluorescent screening assay (Figure 2) using a confocal microscope was employed to track the beads that exhibited: (i) little to no fluorescence when plasma was added; (ii) an increase in fluorescence when bound to the anti-morphine mAb, due to its direct interaction with the illuminating peptide present on the bead; and (iii) a decrease in fluorescence upon the addition of a defined opioid through competitive binding with the antibody. Positive beads (bead C in Figure 2) meeting the desired criteria, i.e., displaying a noticeable change in the fluorescence intensity upon addition of the morphine, were isolated for decoding. The azido-modified illuminating peptides were then synthesized in soluble form and immobilized onto a microarray chip using efficient Cu-free click chemistry, to form a fluorescence-activatable sensor chip which was evaluated for its ability to detect morphine in plasma. 

Peptide microarrays have been used for a variety of research and clinical applications, such as the identification of xenobiotic autoantigens in patients with biliary cirrhosis [30], profiling of cytotoxic T-lymphocyte activity [31], and serodiagnosis of *Burkholderia* infections in cystic fibrosis patients [32]. Unlike our fluorescent-activatable illuminating peptide microarray sensing platform, which utilizes a homogeneous assay, all these microarray platforms use heterogeneous assays that require multiple washing steps. 

In this paper, we reported the discovery of novel morphine-sensitive illuminating peptides using the high throughput OBOC library approach [13] and the development of a fluorescence-based sensor-chip for the detection of morphine in blood. Applying the OBOC platform to the synthesis and detection of MRD-based sensing molecules against morphine is new and unique. This detection platform utilizes a homogeneous immunoassay; therefore, it is fast, simple, and straightforward. In principle, an array of multiple different illuminating peptide sensors can be printed on the chip, such that multiple drugs or disease biomarkers can be detected concurrently in a multiplex manner, using only a minute amount of body fluids, such as blood and urine.

## 2. Results

### 2.1. Selection of the Polymer Beads for Construction of the OBOC Illuminating Peptide Libraries

**Figure 3:**
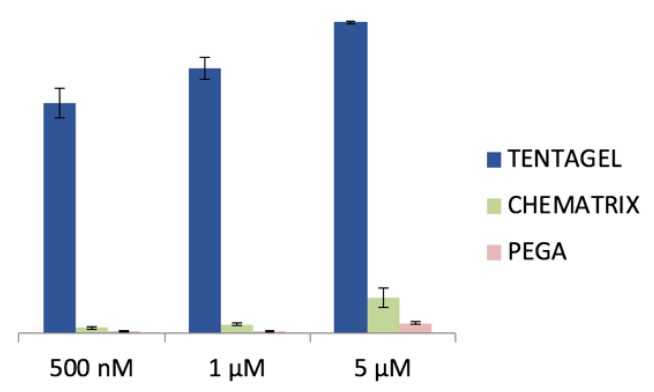
MRD concentrations: 500 nM, 1 μM, and 5 μM. Data collected for six random beads per resin.

To identify an appropriate resin polymer for illuminating peptide discovery, different commercially available beads, including TentaGel, Chematrix, and acrylamide-polyethylene glycol (PEGA) beads were treated with increasing concentrations of MRDs, whilst the dye fluorescent activation was monitored using confocal fluorescence microscopy (CFM) over a period of 1 h. For the PEGA beads, no noticeable activation at the highest MRD concentration (5 μM) was detected (Figure 3). Therefore, to minimize the background fluorescent activation during screening, all the OBOC combinatorial libraries for illuminating peptide discovery were prepared using hydrophilic PEGA beads.

### 2.2. Design and Synthesis of the CEMG

**Figure 4:**
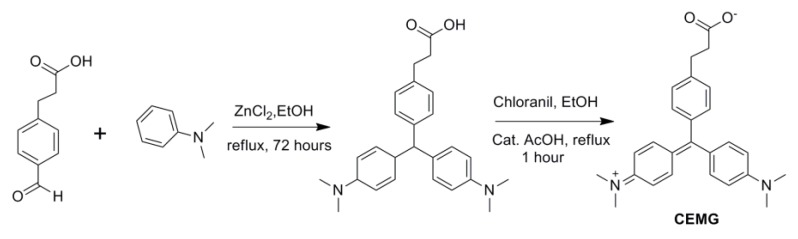
Synthetic scheme of the CEMG.

In order to effectively conjugate the MRDs to the peptide library, we first needed to functionalize the MRD with a carboxyl group. Consequently, CEMG was successfully synthesized in two steps (Figure 4), and its fluorescence excitation-emission spectra were then characterized (Figure 1C). 

### 2.3. Design and Synthesis of the OBOC Combinatorial Peptide Libraries

**Figure 5:**
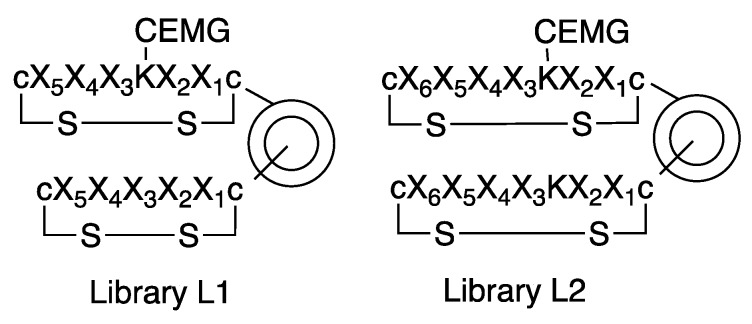
Structures of two OBOC illuminating peptide libraries, where the MRD moiety is introduced within the library onto an amino group of the lysine (K) side chain. X stands for 19 natural amino acids except l-cysteine.

**Figure 6:**
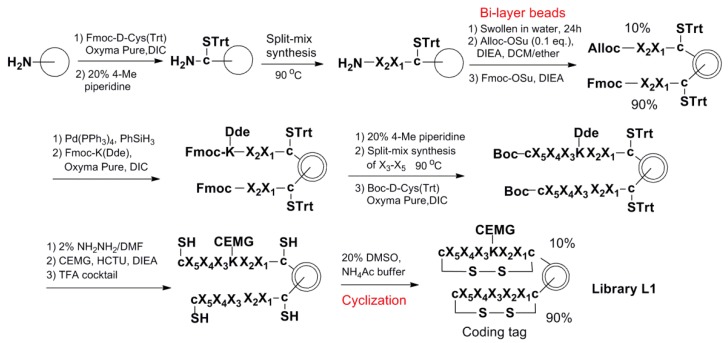
Synthetic scheme of OBOC library L1.

The OBOC combinatorial peptide library method was used to discover the small cyclic illuminating peptides that specifically fluoresce upon binding to an anti-morphine antibody. Two disulfide cyclic OBOC libraries (Figure 5) containing 19^5^ and 19^6^ permutations, respectively, were synthesized on the bi-layer beads [33] via a “split-and-pool” strategy employing fluorenylmethyloxycarbonyl (Fmoc) chemistry [13,34]. In these libraries, the illuminating peptides were displayed on the surface of the beads and the coding tags, without MRD, and they were confined to the bead interior, such that they would not interfere with the screening. In order to speed up the library synthesis, we used the heating method for coupling of the Fmoc-amino acid (90 °C for 2 min) and Fmoc-deprotection (90 °C for 90 s) as described in Reference [35]. The two flanking d-cysteines in the peptides were coupled at room temperature for 2 h to avoid racemization. The synthetic scheme for library L1 is shown in Figure 6 as an example of the library synthesis.

### 2.4. High-Throughput Screening of the OBOC Illuminating Peptide Library Beads

Library beads were successfully immobilized on a polystyrene plate by submersion in 90% *N*,*N*-dimethylformamide (DMF) in water. The plate, containing ~100,000 immobilized beads, (where each displayed a unique illuminating peptide) was scanned within 50 min using a confocal microscope. The library screening was achieved via a three-step strategy as described above and as shown in Figure 2. Changes in the fluorescent intensity (ΔF/F_0_) after each addition were analyzed using a customized algorithm written in MATLAB (Supplemental Figure S1). One strong positive bead from library L1 and three strong positive beads from library L2 were picked up for the Edman microsequencing. The result is shown in Table 1. Some sequence homology was observed between P1 and P2: where both contained 3 tryptophans and an additional hydrophobic amino acid, with two of the tryptophans lined up at the amino side of the Lys(CEMG). The four peptides were resynthesized on the PEGA beads via a similar method as used in the library synthesis. The fluorescence activation of the beads with the anti-morphine mAb and deactivation with the morphine were confirmed with beads P1, P2, and P3 (Supplemental Figure S2). Peptide P4 was excluded in making the sensor chip due to its high background fluorescence. 

### 2.5. Preparation of the Sensor Chip

**Figure 7:**
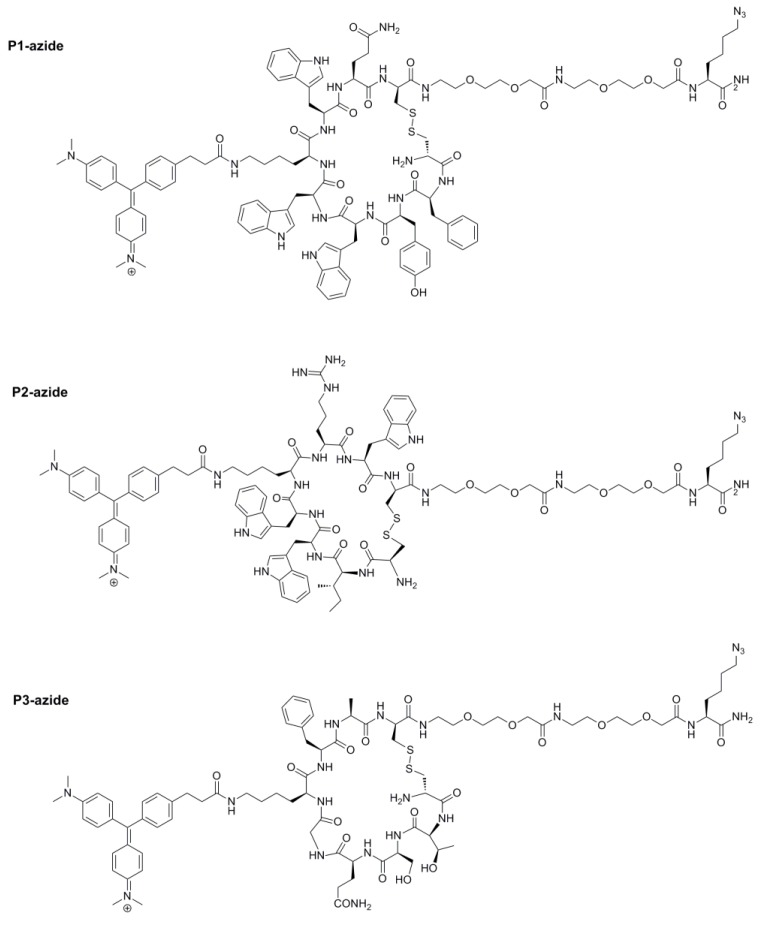
Structures of three azido-illuminating peptides from Table 1.

**Figure 8:**
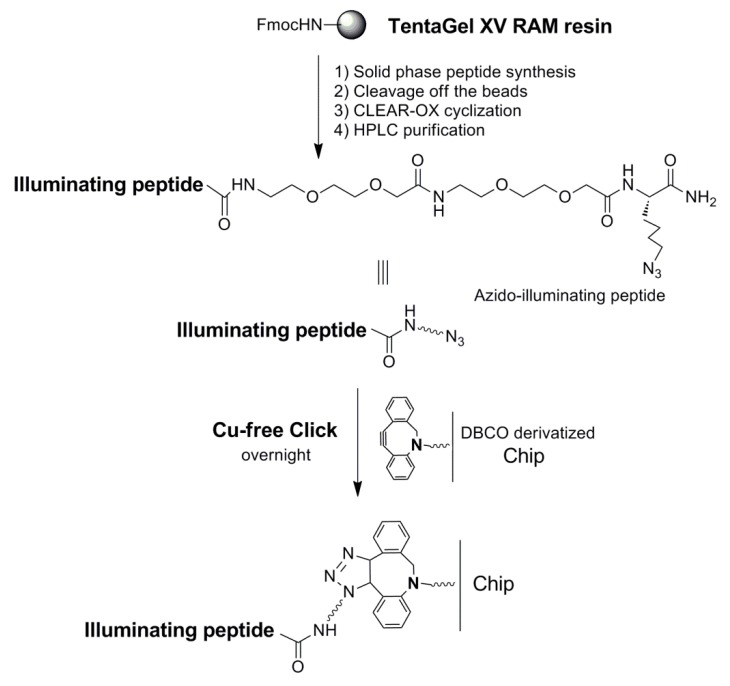
Immobilize illuminating peptides onto a microarray chip using efficient Cu-free click chemistry.

**Figure 9:**
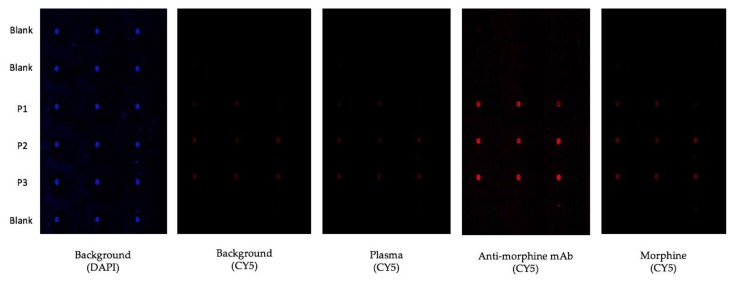
Testing the illuminating peptide sensor chip for morphine detection, after 20 min of incubation. The background picture was taken in PBS. Plasma picture was taken in 10% human blood plasma in PBS. Anti-morphine mAb concentration was 3.5 nM and morphine concentration was 0.35 µM. Rows number 1, 2, and 6 are blank spots without illuminating peptides.

We recently reported the use of photolithography to generate arrays of microdiscs of polymerized PEG functionalized with amino groups for microfluidic assisted in situ peptide print-synthesis of the peptide microarrays [36]. In this study, we utilized the same microdisc arrays as the platform for the sensor chip, but we used the Cu-free dibenzocyclooctyne (DBCO)—azide click chemistry to ligate the illuminating peptides to the chip. DBCO was first introduced to the chip using DBCO-NHS in the presence of DIEA. Three individual azido-illuminating peptides (Figure 7) were synthesized on a TentaGel XV RAM, using the same chemistry used for the OBOC library synthesis. Fmoc-Lys(N_3_)-OH, an unnatural amino acid to introduce an azide group to the peptide, was first coupled to the beads. The linear azido-illuminating peptides were cleaved off the bead, cyclized with CLEAR-OX resins [37,38], and then purified by HPLC. The illuminating peptides were then conjugated to the DBCO-modified micro-chip using the efficient Cu-free click chemistry (Figure 8). The resulting chip was then evaluated for the detection of morphine in human plasma (Figure 9). All three illuminating peptides were found to be able to detect morphine in the plasma at a concentration of 0.35 µM (100 ng/mL).

## 3. Discussion 

We aimed to develop a sensor chip that depended solely on the binding specificity between the anti-opioid mAb and the corresponding illuminating peptides, without the need of washing steps. Such illuminating peptides were easily discovered through the OBOC combinatorial library approach. PEGA beads were chosen as a solid support for the construction of the OBOC libraries because of their low autofluorescence and low propensity to activate MG. MRD CEMG was used to construct the OBOC illuminating peptide libraries because of its ability to be easily conjugated to the lysine side chain of the peptide sequence. 

The OBOC libraries were synthesized on bi-layer beads wherein the coding tag resided inside the bead to avoid interference with the screening probe [33]. In order to speed up the library synthesis, we used the heating method for coupling of the Fmoc-amino acid and Fmoc-deprotection [35]. To the best of our knowledge, this is the first report that applied the heating method to the synthesis of the OBOC library. Each library contains millions of beads, where each bead displays a unique illuminating peptide. The disulfide cyclic library yields conformational constrained peptides, which provide an ideal molecule for MRD activation upon the antibody binding. The simple method for immobilizing the library beads on polystyrene plates [39,40] enabled us to perform a sequential three-step screening, such that the fluorescent image of each and every library bead could be easily tracked and recorded. Beads that fulfilled the following three criteria were considered positive beads: (i) They did not fluoresce in the presence of plasma, (ii) they fluoresced when the anti-morphine mAb was added, and (iii) the fluorescent signal diminished greatly upon addition of the free morphine to the screening medium. Of the four positive beads isolated (Table 1), only three of them (P1, P2, and P3) were confirmed to be true positive upon resynthesis of the peptides on the PEGA beads (Supplemental Figure S2). P1 and P2 had significant sequence homology. Based on the homology, focused OBOC libraries can be developed to discover more sensitive illuminating peptides for morphine. Without further optimization, the three illuminating peptides can already easily detect 0.35 μM of morphine in the plasma. The lethal intoxication concentration of free morphine in the blood is about 170 ng/mL (median, equal to 0.6 μM) [41]. For other opioid drugs, such as fentanyl, which is much more potent and has lower blood and urine levels in addicts, their detection will require more sensitive fluorescent-activating peptides. Since plasma exposure was incorporated into one of the early screening steps, and the beads that fluoresced in plasma were eliminated from further consideration, the identified illuminating peptides were free of interference by the plasma proteins.

The antibody-based homogeneous assay reported here, after optimization, can potentially be used as an inexpensive point-of-care test for instant detection, identification, and quantification of specific opioids in bodily fluids. Although morphine was used as an example, the illuminating peptides against other opioid drugs and other street drugs (e.g., amphetamines) can also be discovered using this novel approach. We envision that the illuminating peptides against a range of controlled substances can be printed on microarrays for the concurrent screening of a large number of drugs. 

It is noteworthy that during the course of the screening, we found a few beads that did not fluoresce in neither the plasma nor the addition of anti-morphine mAb but fluoresced upon the addition of morphine. This indicated that these cyclic peptides interacted with morphine directly. Work is currently underway to characterize these peptides. If interaction is specific and with high affinity, these morphine sensing peptides could potentially be used to develop an inexpensive sensor chip for the direct detection of opiates without the use of antibodies.

In summary, we have successfully developed a morphine-sensitive sensor chip and demonstrated that this proof-of-concept platform can be used to develop fluorescence-based sensors against morphine in a straightforward homogeneous immunoassay. The ultimate application of this platform is to develop a point-of-care device using a portable smartphone-based fluorescent reader (e.g., DxCELL fluorescent reader), for the instant and sensitive detection of opioids in body fluids, which is not currently available in the market. This fluorescent-activating peptide concept can potentially be applied to the development of sensor chips against not only illicit drugs, but also biomarkers for cancer and other diseases.

## 4. Materials and Methods 

### 4.1. General Experiment Procedures 

The OBOC library screening was done on a confocal microscope ZEISS LSM 800 (Carl Zeiss Microscopy, Thornwood, NY, USA). Data analysis was conducted using the Fiji and MATLAB programs. Matrix-assisted laser desorption/ionization time of flight mass spectrometry (MALDI-TOF MS) analysis was performed on a Bruker UltraFlextreme mass spectrometer (Billerica, MA, USA). The analytical HPLC was performed on a Waters 2996 HPLC system equipped with a 4.6 × 150 mm Waters Xterra MS C18 5.0 µm column, and it employed a 20 min gradient from 100% aqueous H_2_O (0.1% TFA) to 100% CH_3_CN (0.1% TFA), at a flow rate of 1.0 mL/min. Preparative HPLC was performed on a System Gold 126NMP solvent module (Beckman), with a C18 column (Vydac, 10 µm, 2.2 cm i.d. × 25 cm). A gradient elution of 0–60% B over 45 min, then 60–100% B over 5 min, followed by 100% B for 5 min, was used at a flow rate of 5 mL/min (solvent A, H_2_O/0.1% TFA; B, acetonitrile/0.1% TFA).

PEGA beads (0.4 mmol/g, 150–300 µm) were purchased from Agilent Technologies (Santa Clara, CA, USA). TentaGel XV RAM resin (0.28 mmol/g) was purchased from Rapp Polymere GmbH (Tϋbingen, Germany). 1-[Bis(dimethylamino)methylene]-1*H*-1,2,3-triazolo[4,5-b]pyridinium 3-oxid hexafluorophosphate (HATU) and Fmoc-amino acids were purchased from P3BioSystems (Louisville, KY, USA). CLEAR-OX^TM^ resin was purchased from Peptide International Inc. (Louisville, KY, USA). *O*-(1*H*-6-Chlorobenzotriazole-1-yl)-1,1,3,3-tetramethyluronium hexafluorophosphate (HCTU), Boc-D-Cys(Trt)-OH and ethyl cyanohydroxy-iminoacetate (Oxyma Pure) were purchased from Chem-Impex International Inc. (Wood Dale, IL, USA). Fmoc-Lys(Dde)-OH was purchased from AAPPTec (Louisville, KY, USA). Fmoc-AEEA linker and Fmoc-Lys(N_3_)-OH were purchased from ChemPep Inc. (Wellington, FL, USA). DBCO-NHS was obtained from BroadPharm (San Diego, CA, USA). 1,3-Diisopropylcarbodiimide (DIC), trifluoroacetic acid (TFA), *N*,*N*-diisopropylethylamine (DIEA), triisopropylsilane (TIS), dimethyl sulfoxide (DMSO), morphine sulfate salt pentahydrate, all solvents, and other chemical reagents were purchased from Sigma-Aldrich (St. Louis, MO, USA), and they were of analytical grade. The anti-morphine monoclonal antibody was purchased from MyBioSource (San Diego, CA, USA).

### 4.2. Synthesis of the CEMG

Under an atmosphere of nitrogen, 0.3 g of 3-(4-formylphenyl)propanoic acid (1.7 mmol, 1 eq) and 0.5 g of ZnCl_2_ (3.7 mmol, 2.2 eq) were added in 18 mL of anhydrous ethanol. Then, 0.47 mL of *N*,*N*-dimethylaniline (3.7 mmol, 2.2 eq) was injected into the reaction mixture, which was allowed to stir under reflux overnight. The solvent was removed under reduced pressure and the crude product was purified by flash column chromatography, using a gradient from 0–5% methanol (MeOH) in dichloromethane (DCM) to yield 0.51 g (y = 75%) of leucomalachite green as a pale green solid. 1H NMR (400 MHz, CDCl_3_): δ 7.07 (4H, q, *J* = 8.0 Hz), 6.97 (4H, d, *J* = 8.5 Hz), 6.71–6.62 (4H, d, *J* = 8.4 Hz), 5.34 (1H, s), 2.93 (2H, t, *J* = 8.6 Hz), 2.90 (12H, s), 2.65 (2H, t, *J* = 7.9 Hz). ^13^C-NMR (101 MHz, CDCl_3_): δ 178.33, 148.82, 143.26, 137.70, 133.34, 129.84, 129.37, 127.92, 112.98, 54.62, 40.90, 35.67, 30.24. HRMS (ESI): [M + H]^+^ *m*/*z* calcd 403.2380, found 403.2371.

Thereafter, 0.30 g of leucomalachite green was dissolved in 15 mL of hot 95% ethanol (EtOH) in a reaction flask. Then, 0.275 g (1.5 eq.) of chloranil and a catalytic amount of acetic acid were added to the EtOH solution, and the mixture was refluxed for 1 h. The solution turned dark green within the first few minutes. The solvent was removed under vacuum and the residue was purified by flash column chromatography using a gradient from 0 to 6% MeOH in DCM to yield 245 mg (y = 82%) of CEMG zwitterion. ^1^H-NMR (400 MHz, CDCl_3_): δ 7.42 (2H, d, *J* = 7.8 Hz), 7.36 (4H, d, *J* = 8.9 Hz), 7.17 (2H, d, *J* = 7.8 Hz), 6.91 (4H, d, *J* = 8.9 Hz), 3.34 (12H, s), 3.08 (2H, t, *J* = 7.5 Hz), 2.96 (2H, t, *J* = 7.5 Hz). ^13^C-NMR (101 MHz, CDCl_3_): δ 178.64, 174.26, 156.79, 148.33, 141.00, 137.02, 135.16, 129.12, 127.19, 113.49, 41.00, 36.03, 31.15. HRMS (ESI): [M + H]^+^ *m*/*z* calcd 401.2224, found 401.2220.

### 4.3. Synthesis of the OBOC Libraries

Figure 6 describes the library synthesis scheme. One gram of PEGA resin beads were swollen in DMF for 1 h before washing with DMF, MeOH, and DMF. Fmoc-D-Cys(Trt) was coupled to the beads in the presence of Oxyma Pure and DIC using 6 eq excess. The coupling proceeded at room temperature for 2 h. After the liquid was drained, the beads were washed with DMF, MeOH, and DMF. Fmoc-deprotection was achieved using 20% 4-methyl piperidine in DMF, for 5 min, and then 15 min. The beads were washed and separated into 19 aliquots, each aliquot was reacted with *6*-fold molar excess of one of 19 Fmoc-amino acids (X_1_). Coupling was initiated by the addition of 6 eq excess of Oxyma Pure and DIC. The coupling reactions were performed at 90 °C for 2 min as in Reference [35] in a 20-well heating block, and then monitored using the ninhydrin test. After the liquid was drained, the beads were washed with DMF and then deprotected using 20% 4-methyl piperidine in DMF at 90 °C for 90 s in a 20-well heating block. The beads were then combined and washed with DMF, MeOH, DMF, and re-divided for the next cycle of coupling (X_2_). After the beads were combined and Fmoc-deprotected, the beads were washed with DMF, MeOH, and DCM. The beads were then dried in vacuum. The bi-layer beads were prepared using our previous bi-phasic solvent approach [33]. Then, the beads were swollen in water for 24 h. The water was removed by filtration, and the solution of Alloc-OSu (0.1 eq to the bead loading) in DCM/diethyl ether (*v*/*v*, 55/45) mixture was added to the wet beads, followed by the addition of DIEA. The mixture was shaken vigorously at room temperature for 45 min. After removal of the liquid by filtration, the beads were washed with DMF to remove water from inside the beads, followed by MeOH and DMF. A solution of Fmoc-OSu (3 eq) and DIEA (6 eq) in DCM was then added to the beads. The beads were shaken at room temperature for 1 h. Alloc was deprotected with Pd(PPh_3_)_4_ (0.2 eq) and PhSiH_3_ (20 eq) in DCM, for 45 min twice. Following de-protection, the beads were washed sequentially with DCM, DMF, 0.5% DIEA in DMF, 0.5% sodium diethyldithiocarbamate in DMF, 50% DCM in DMF, MeOH, and DMF. Fmoc-Lys(Dde)-OH was coupled to the outer of beads using Oxyma Pure and DIC. The Fmoc on both the outer layer and inner layer was removed as described above. After the beads were washed, the beads were then used for the remaining cycles of split-and-pool synthesis of X_3_–X_6_ using the heating method. The Boc-D-Cys(Trt)-OH was coupled at the last cycle of the peptide assembling using HCTU and DIEA at room temperature for 2 h, until the ninhydrin test was negative. Then, Dde was removed with 2% hydrazine in DMF at room temperature, 3 times, each time for 3 min. After washing with DMF, MeOH, and DMF, the CEMG was coupled to the beads in the presence of HATU and DIEA overnight. After thorough washing with DMF, MeOH, and DCM, the beads were dried in vacuum. The side chain protecting groups were removed with a cocktail containing phenol/thioanisole/water/TIS/TFA (7.5:5:5:2.5:82.5, *w*/*v*/*v*/*v*/*v*). The cleavage solution was drained, and the bead-supported library was washed with DMF, MeOH, DCM, DMF, 50% DMF/water, and water. The libraries were then cyclized via disulfide bond linkage of the two flanking d-cysteines in 500 mL oxidation solution: water/acetic acid/DMSO (75:5:20, *v*/*v*/*v*), where the pH was adjusted to 6 with ammonium hydroxide before adding DMSO, for 2–3 days. Finally, the library beads were washed thoroughly with water, 50% DMF/water, DMF, and then stored in 90% DMF/water. 

### 4.4. High-Throughput Screening of the Immobilized OBOC Illuminating Peptide Libraries

The library beads were suspended in 90% DMF/water, and then added onto a polystyrene plate (120 mm × 80 mm) and allowed to sit still for 30 min. Afterwards the DMF solution was gently removed with a pipette and the immobilized library beads were washed with 50% DMF/water, water, PBS, and then incubated with 10% plasma in PBS for 20 min, followed by scanning with a confocal fluorescence microscope. The fluorescent activation from the peptide bound to plasma proteins was then recorded. Next, the plate was incubated with 3.5 nM of anti-morphine antibody for 20 min, and the fluorescent activation from the illuminating peptide bound antibody was monitored. Thereafter, 0.35 µM morphine solution was added to the plate followed by measurement of the fluorescent intensity. Changes in the fluorescent intensity (ΔF/F_0_) after each addition were analyzed using a customized algorithm written in MATLAB. Then, positive beads were selected based on the largest fluorescence changes, and they were retrieved for decoding using Edman microsequencing.

### 4.5. Resynthesis of the Azido-Illuminating Peptides in Soluble Form

The three azido-illuminating peptides (Figure 7) were synthesized in soluble form using TentaGel XV RAM resin beads. After removal of the Fmoc from the beads with 20% 4-methyl piperidine in DMF (for 5 min, and then 15 min), Fmoc-Lys(N_3_)-OH and two AEEA were coupled using 5 molar excess of [2-(2-(Fmoc-amino)ethoxy)ethoxy]acetic acid (Fmoc-AEEA-OH), Oxyma pure and DIC in DMF, respectively. The coupling reaction was conducted at room temperature for 2 h and monitored using the ninhydrin test. The resin was then washed, and deprotected using 20% 4-methyl piperidine in DMF at room temperature (for 5 min, and then 15 min). They were then washed again, and the first step was repeated to couple the second AEEA spacer. The coupling and deprotection cycles were repeated until the desired cycles of coupling were completed. The CEMG was then coupled to the lysine side chain after Dde deprotection as described in the library synthesis. To cleave the peptide from the resin, as well as to remove the side chain protecting groups, the resins were soaked in the cleavage solution, phenol/thioanisole/water/TIS/TFA (7.5:5:5:2.5:82.5, *w*/*v*/*v*/*v*/*v*) for 4 h. The liquid was then collected and the peptide was precipitated with cold diethyl ether. The disulfide bridge was then successfully made using CLEAR-OX resins as in Reference [37] and the peptides were purified using reversed phase HPLC. The purity was determined to be >90%. The identity of the compounds was confirmed using the MALDI-TOF MS. P1 *m/z* calcd 2174.00, found 2175.25; P2 *m/z*: calcd 2004.99, found 2004.97; P3 *m/z*: calcd 1767.83, found 1767.67.

### 4.6. Preparation of Sensor-Chip and Testing for Morphine-Binding 

The PET sheet was chemically modified using silane coating, which allowed PDMS polymerization on the PET surface to support covalent bond formation. The silane modified PET was then treated with oxygen plasma to form hydroxy groups on the surface, and then uncured PDMS was spin coated onto the PET sheet and it underwent polymerization in a heated oven. Microdisc carriers were fabricated using our published photolithography technology [36]. Polyethylene glycol (PEG) was chosen as the structural material due to its long-term biocompatibility, ability to conjugate other functional derivatives, optical clarity, and most importantly, its excellent swelling property in both polar and non-polar solvents. The polymer composite included: 200 μL of PEG-diacrylate (MW 700 Da), 112 μL of trimethylolpropane ethoxylate triacrylate (cross-linker), 8 μL of 2-hydroxyl-2-methylpropiophenone (photo initiator), and 300 μL of 2-aminoethyl methacrylate·HCl (7.2 mg) solution in deionized water. Under ultraviolet exposure, the acrylate molecules can be polymerized to form a highly insoluble matrix. The addition of 2-aminoethyl methacrylate hydrochloride forms an amine-terminated end group of the matrix for the subsequent peptide synthesis [36]. Then, two Fmoc-AEEA spacers were coupled to the free amine groups on the chip using HCTU/DIEA coupling, following by Fmoc-deprotection and the addition of DBCO-OSu in the presence of DIEA. Illuminating peptides were dissolved in DMF, and then added to the corresponding spots on the chip and incubated at 4 °C overnight. After thorough washing with DMF, MeOH, DMF, 50% DMF/H_2_O, and H_2_O, the chip was ready for testing.

The chip was then placed in a polystyrene plate and PBS was added to the plate to cover all the microdiscs on the chip. Then, the chip was scanned by a confocal microscope to obtain the background image (Figure 9-background). To eliminate the non-specific positive hits, it was then incubated with 10% plasma in PBS for 20 min and the fluorescent activation from the illuminating peptide bound plasma proteins was monitored using a confocal microscope (Figure 9-plasma). The chip was then incubated with 3.5 nM of anti-morphine mAb for 20 min, and the fluorescent activation from the illuminating peptide bound antibody was monitored (Figure 9-anti-morphine mAb). Finally, 0.35 µM morphine solution was added to the plate and it was incubated for 20 min, followed by measurement of the fluorescent intensity (Figure 9-morphine).

## Figures and Tables

**Table 1:** Sequences of cyclic positive illuminating peptide hits.

	c	X_6_	X_5_	X_4_	X_3_	K(CEMG)	X_2_	X_1_	c
**P1**	c	F	Y	W	W	K(CEMG)	W	Q	c
**P2**	c		I	W	W	K(CEMG)	R	W	c
**P3**	c	T	S	Q	G	K(CEMG)	F	A	c
**P4**	c	N	V	G	N	K(CEMG)	Q	P	c

## Supplementary Materials

The following are available online at. Figure S1, Automated analysis of the illuminating peptide library beads; Figure S2, Binding confirmation of the resynthesized illuminating peptides on beads.
