# Supplementary material for: Rapid Discovery of Illuminating Peptides for Instant Detection of Opioids in Blood and Body Fluids
Source: Molecules. 2019 May 10;24(9):1813. doi: 10.3390/molecules24091813 (PMC6539258; doi:10.3390/molecules24091813)
Supplement: Supplementary file 1 [file molecules-24-01813-s001.pdf]

Supplemental Figure S1

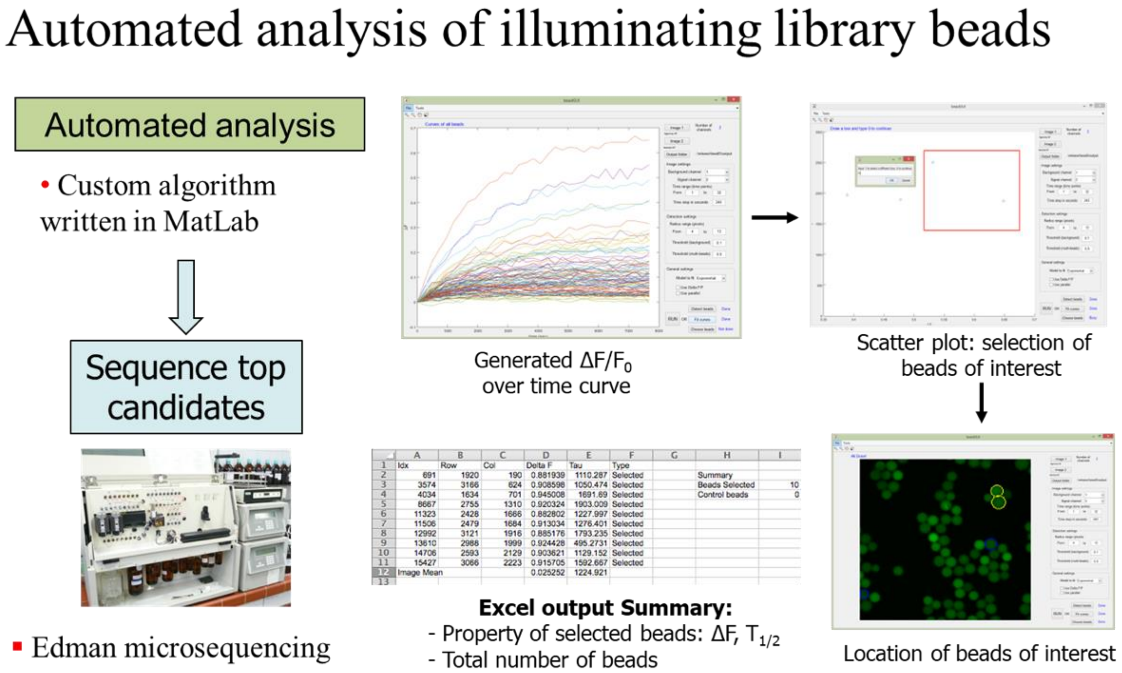

Supplemental Figure S2

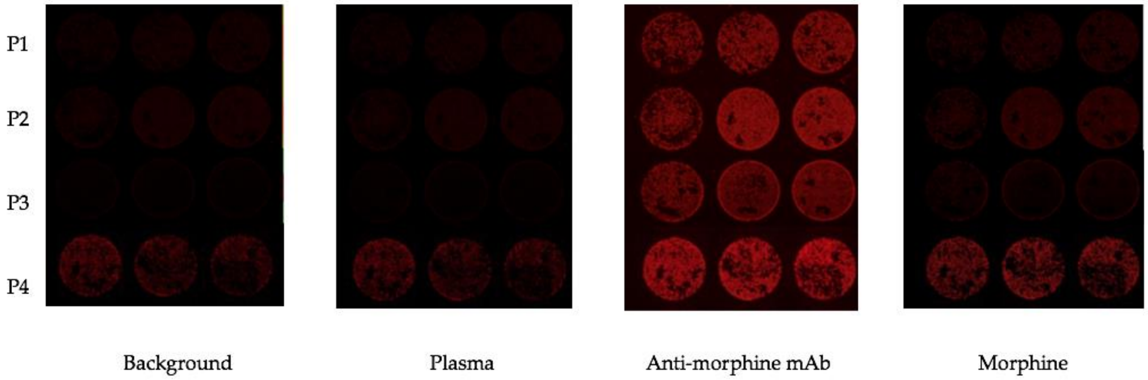

Binding confirmation of resynthesized illuminating peptides on beads. Peptide-beads were immobilized and tested in a 48-well plate. The incubation time of beads with plasma, anti-morphine mAb and free morphine is 20 minutes. Background picture was taken in PBS. Plasma picture was taken in 10% human plasma in PBS. Anti-morphine MAb concentration was 3.5 nM and morphine concentration was 0.35 μM.
